# Modeling the Stability of SARS-CoV-2 on Personal Protective Equipment (PPE)

**DOI:** 10.4269/ajtmh.20-1508

**Published:** 2020-12-22

**Authors:** Andrew D. Haddow, Taylor R. Watt, Holly A. Bloomfield, David P. Fetterer, David E. Harbourt

**Affiliations:** 1 General Dynamics Health Solutions in Support of USAMRIID, Fort Detrick, Maryland;; 2 United States Army Medical Research Institute of Infectious Diseases (USAMRIID), Fort Detrick, Maryland;; 3 ICON Global Public Health Solutions, Fort Detrick, Maryland

## Abstract

We modeled the stability of SARS-CoV-2 on personal protective equipment (PPE) commonly worn in hospitals when carrying out high-risk airway procedures. Evaluated PPE included the visors and hoods of two brands of commercially available powered air purifying respirators, a disposable face shield, and Tyvek coveralls. Following an exposure to 4.3 log_10_ plaque-forming units (PFUs) of SARS-CoV-2, all materials displayed a reduction in titer of > 4.2 log_10_ by 72 hours postexposure, with detectable titers at 72 hours varying by material (1.1–2.3 log_10_ PFU/mL). Our results highlight the need for proper doffing and disinfection of PPE, or disposal, to reduce the risk of SARS-CoV-2 contact or fomite transmission.

Transmission of SARS-CoV-2, the causative agent of COVID-19, occurs primarily through respiratory droplets in close contact settings or by airborne transmission in poorly ventilated enclosed spaces.^1^ Contact transmission, also known as fomite transmission, occurs through contact with contaminated materials or surfaces. Personal protective equipment (PPE) worn by healthcare providers is exposed to SARS-CoV-2 while caring for infectious patients, in turn increasing the chances of fomite transmission of the virus during PPE doffing or reuse without prior disinfection. Certain high-risk airway procedures such as intubation or suctioning have the potential to produce large amounts of phlegm, mucus, and/or saliva, in addition to aerosols.^2^ Thus, PPE worn by healthcare workers when performing these or similar procedures are exposed to higher virus concentrations than those worn during lower-risk medical procedures not involving the patient’s airway.^3^ Herein, we carried out a pilot study to model the stability of SARS-CoV-2 on common PPE worn in hospitals to simulate a moderate-dose SARS-CoV-2 transmission event that might occur during a high-risk airway procedure on a COVID-19 patient.

Evaluated materials comprised 6.3 mm^2^ of selected PPE (Table 1). We also evaluated 50/50 nylon/cotton ripstop fabric treated with insect shield (permethrin 0.5% [%W/W]: Tullahoma Industries, LLC, Tullahoma, TN). All materials were surface disinfected by ultraviolet (UV) light for 10 minutes, after which they were contained within covered six-well cell culture plates to mimic PPE storage, and incubated at 22 ± 2°C with a relative humidity of 40–50% in an operating class II biosafety cabinet throughout the duration of the experiment. Triplicate samples of each material were exposed to 50 µL of SARS-CoV-2 (USA-WA1/2020, GenBank accession no. MN985325.1), with the challenge dose being 4.3 log_10_ plaque-forming units (PFUs). This dose was selected based on reported viral RNA loads in sputum of human clinical samples.^4^ Samples were collected at 4, 8, 24, 48, and 72 hours postexposure. A 72-hour time frame was selected as it coincides with a common work schedule observed by many intensive care unit personnel (e.g., nurses) in the United States (three 12-hour shifts worked over three consecutive days of the week) who may need to reuse PPE day after day because of PPE shortages. Forceps were used to collect samples and place them into 2 mL tubes containing media as previously described,^5^ after which forceps were disinfected between samples using 5% MicroChem™ Plus followed by 70% ethanol. Samples were then stored at −80°C before quantification of infectious virus. Virus titration was performed in duplicate via plaque assay on Vero 76 cells (ATCC, Manassas, VA; CRL-1587) as previously described,^5^ with the limit of detection being 1.0 log_10_ PFU/mL.

**Table 1:** Personnel protective equipment evaluated and associated geometric mean half-life in hours by material

Product	Manufacturer	Material	Material description	Geometric mean half-life* (95% CI)
3M Versaflo economy hood	3M (Cat No. S-403)	Visor	Polyethylene terephthalate glycol	10.05 (9.496–10.642)
		Shroud fabric	Polypropylene-coated nonwoven polypropylene	9.12 (8.444–9.858)
ILC Dover SENTINEL XL BioShield full hood	ILC Dover (Cat No. S-2028)	Visor	Optically clear polyester	8.72 (7.615–9.981)
		Shroud fabric	Spunbound polypropylene nonwoven with a polyethylene outer film	6.74 (5.639–8.051)
FisherBrand disposable full face shield antifog	ThermoFisher Scientific (Cat No. 19-460-102)	Visor	Polyester treated with an antifog and antistatic coating	8.83 (7.383–10.554)
DuPont Tyvek 400 coverall	DuPont (Cat No. TY127SWH)	Fabric	DuPont Tyvek 400	9.08 (7.635–10.802)

* Hours.

Half-lives were estimated by fitting a Poisson regression to each sample, and *t*-statistic CIs were computed on the log half-lives. Predicted mean titers were based on an over-dispersed Poisson generalized estimating equation. Analyses were implemented in SAS version 9.4 (SAS Institute Inc., Cary, NC).

We found an inverse relationship between SARS-CoV-2 stability on material surfaces and time (Figure 1), as reported in previous studies.^5–15^ At 72 hours postexposure, all PPE materials had similar detectable titers, with the exception of the 3M™ Versaflo™ economy hood (shroud fabric), Saint Paul, MN, which had a titer of 1.1 log_10_ PFU/mL. By 72 hours postexposure, all materials displayed a reduction in titer ≥ 4.29 log_10_, with final titers of 2.3 log_10_ PFU/mL (3M Versaflo economy hood visor), 1.9 log_10_ PFU/mL (ILC Dover SENTINEL XL^®^ BioShield full hood visor, ILC Dover, Frederica, DE), 2.2 log_10_ PFU/mL (FisherBrand^®^ disposable full face shield antifog, ThermoFisher Scientific, Waltham, MA), 2.1 log_10_ PFU/mL (3M Versaflo economy hood shroud fabric), and 2.1 log_10_ PFU/mL (DuPont™ Tyvek^®^ 400 Coverall, Midland, MI). The geometric mean half-life of all PPE materials varied from between 6.74 and 10.05 hours (Table 1 and Supplemental Figure 1), with the ILC Dover SENTINEL XL^®^ BioShield full hood visor and shroud fabric displaying the shortest half-life. Viable virus on the Insect Shield treated 50/50 nylon/cotton ripstop fabric decreased rapidly, 2.8 log_10_ PFU/mL at 4 hours postexposure, and 2.1 log_10_ PFU/mL at 8 hours postexposure, and by 24 hours postexposure, no viable virus was detected, with the geometric mean half-life being 0.90 hours (95% CI: 0.645–1.249).

**Figure 1.**
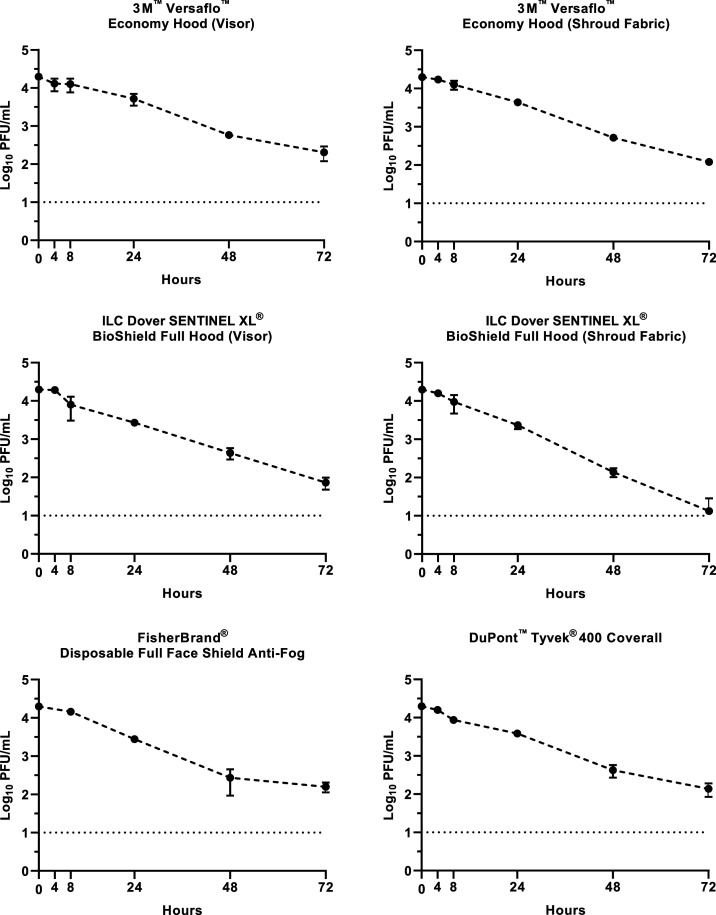
Detection of infectious virus on personal protection equipment following a SARS-CoV-2 exposure. No data are reported for the FisherBrand disposable full face shield antifog at 4 hours postexposure as all three samples were inadvertently flipped onto their exposed surface because of a static charge while transferring. The lower limit of detection was 1.0 log_10_ plaque-forming unit (PFU)/mL.

SARS-CoV-2 displayed prolonged stability on those PPE materials evaluated in this study, all of which had infectious SARS-CoV-2 present at least 72 hours postexposure. A recent preprint reported virus recovery at 14 days postexposure on the plastic visor of the 3M Model BE-10L powered air-purifying respirators hood, and at 21 days postexposure on DuPont Tyvek 400 using stabilized virus containing mucin, bovine serum albumin, and tryptone,^15^ whereas our study used virus in media. Although we found the predicted half-life of SARS-CoV-2 on PPE ranged from 6.74 to 10.05 hours (depending on the PPE material), it is important to note that between 1.9 and 2.3 log_10_ PFU/mL of infectious virus remained on those PPE evaluated in this study at 72 hours postexposure. This is in contrast to SARS-CoV-2 viability on cloth fabric (i.e., 50/50 nylon/cotton ripstop fabric), which decreased rapidly and was not recoverable at 24 hours postexposure, as reported elsewhere.^5,15^

The results of this study should be interpreted in light of its limitations. Although the use of six-well tissue culture plates to hold exposed materials allowed air exchange, their use may have reduced virus desiccation. In addition, although exposed materials were not stored in darkness, continuous light was not always present. Similarly, the optical coating on the tissue culture plates and on the biosafety cabinet sash would have limited exposure to UV light reducing virus degradation. We exposed the materials in this study to a moderate dose of SARS-CoV-2 in 50 µL of media. Although certain high-risk airway procedures could result in such virus doses/volumes, most healthcare providers PPE would likely receive lower doses of SARS-CoV-2 while caring for COVID-19 patients. Thus, future work is needed to evaluate the stability of SARS-CoV-2 on PPE modeling transmission via infectious respiratory droplets (> 5 µm) and/or droplet nuclei (≤ 5 µm) in an effort to model the likelihood of fomite transmission via PPE contaminated in such a manner.

In summary, we demonstrated that SARS-CoV-2 remained infectious on some types of healthcare PPE for at least 72 hours postexposure at 22 ± 2°C following a moderate-dose exposure designed to mimic virus concentrations that might be achieved during high-risk airway procedures. Although our results highlight the stability of SARS-CoV-2 on PPE in an experimental setting, proper doffing and/or disposal or disinfection of PPE would reduce the likelihood of fomite transmission following such procedures.

## Supplemental figure

Supplemental materials
