# Supplementary material for: Modeling the Stability of SARS-CoV-2 on Personal Protective Equipment (PPE)
Source: Am J Trop Med Hyg. 2020 Dec 22;104(2):549–51. doi: 10.4269/ajtmh.20-1508 (PMC7866333; doi:10.4269/ajtmh.20-1508)
Supplement: Supplementary file 1 [file tpmd201508.SD1.pdf]

Supplementary Figure.

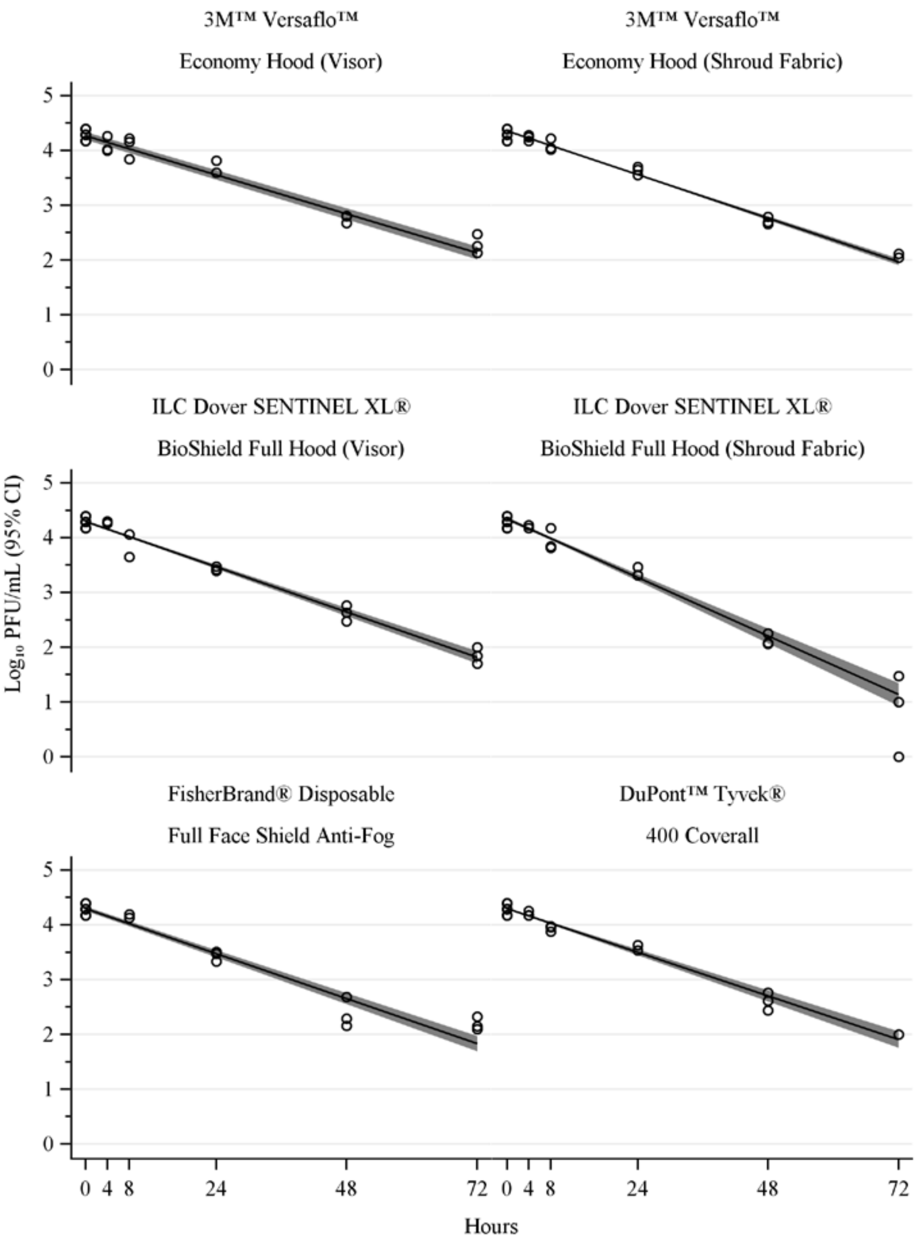

Geometric mean half-life of SARS-CoV-2 on Personal Protection Equipment (PPE). The line indicates the estimated mean titer, with bands extending to the 95% Confidence Interval. Estimates are based on an over-dispersed Poisson generalized estimating equation. Points indicate individual observations. Observations of 0 PFU are plotted at 0.01 PFU/mL.
